# The More Publication, the Higher Impact Factor: Citation Analysis of Top Nine Gastroenterology and Hepatology Journals

**DOI:** 10.5812/hepatmon.8467

**Published:** 2012-12-30

**Authors:** Pegah Karimi Elizee, Romina Karimzadeh Ghassab, Azam Raoofi, Seyyed Mohammad Miri

**Affiliations:** 1 Kowsar Corporation, Hoensbroek, The Netherlands; 2 Baqiyatallah Research Center for Gastroenterology and Research Center, Tehran, IR Iran

**Keywords:** Self-Citation, Journal Impact Factor, Gastroenterology

## Abstract

**Background:**

The impact factor (IF), as the most important criterion for journal’s quality measurement, is affected by the self-citation and number of publications in each journal.

**Objectives:**

To find out the relationship between the number of publications and self-citations in a journal, and their correlations with IF.

**Materials and Methods:**

Self-citations and impact factors of nine top gastroenterology and hepatology journals were assessed during the seven recent years (2005-2011) through Journal Citation Reports (JCR, ISI Thomson Reuters).

**Results:**

Although impact factors of all journals increased during the study, five out of nine journals increased the number of publications from 2005 to 2011. There was an increase in self-citation only in the journal of HEPATOLOGY (499 in 2005 vs. 707 in 2011). Impact factors of journals (6.5 ± 3.5) were positively correlated with total number of publications (248.6 ± 91.7) (R: 0.688, P < 0.001). Besides, the self-citation rate (238.73 ± 195.317) was highly correlated with total number of publications in each journal (248.6 ± 91.7) (R: 0.861, P < 0.001). On the other hand, impact factor without self-citation (6.08 ± 3.3) had a correlation (R: 0.672, P < 0.001) with the number of published items (248.6 ± 91.7).

**Conclusions:**

The number of articles and self-citation have definite effects on IF of a journal and because IF is the most prominent criterion for journal’s quality measurement, it would be a good idea to consider factors affecting on IF such as self-citation.

## 1. Background

Originally, impact factor (IF) was introduced by ISI Web of Science, and was an indicator for the number of citations to the published articles in a journal during the two preceding years. Recently, IF is known as a significant scientometric parameter of journal’s value: the higher an impact factor, the more qualified a journal (1). However, it could not be considered as the best tool for measuring journals’ quality (2, 3). Some studies revealed several factors that might play important roles in IF, including field of a journal, article type, number of contributors, number of published articles in a journal (4), and eventually self-citation (5) which means citing to a reference from an identical journal. Self-citation constitutes a natural part of a journal’s IF (6). Somehow, self-citation rate may reflect the field of a journal (7, 8): the higher self-citation rates, the more isolated or narrower fields. This is in contrast to multidisciplinary journals which constitute fewer self-citations (7). Due to the potential roles of self-citation in IF calculation, the editors should be aware of advantages and disadvantages of this parameter in the quality evaluation of journals (9).

## 2. Objectives

Presuming the effects of self-citation on enhancing the journals’ IF, we aimed to evaluate the relationship between self-citation, number of publications, and IF during seven years among top nine gastroenterology and hepatology journals indexed in ISI.

## 3. Materials and Methods

In the present study, the self-citations and impact factors of nine top gastroenterology and hepatology journals as well as the number of their publications during the seven recent years (2005-2011) were assessed (Table 1 and 2). Data were collected from Journal Citation Reports (JCR, ISI Thomson Reuters). IF calculation was performed based on the standard formula (10) by dividing the number of citations in each year to the articles published in the journal during the two preceding years to the total number of published articles in the journal. The relationships of the number of published articles with IF and self-citation were also analyzed using SPSS version 16, and P value less than 0.05 was considered as significant; also, Microsoft Excel 2010 was used for drawing graphs. The linear regression analysis and Pearson correlation coefficient were used to evaluate the correlation between two factors.

**Table 1:** List of Nine Top Gastroenterology and Hepatology Journals

Full Journal Title	JCR Abbreviation Title	ISSN
**Gastroenterology**	Gastroenterology	0016-5085
**Hepatology**	Hepatology	0270-9139
**GUT**	GUT	0017-5749
**Journal of Hepatology**	J Hepatol	0168-8278
**American Journal of Gastroenterology**	AM J Gastroenterol	0002-9270
**Liver International**	Liver Int	1478-3223
**Journal of Gastroenterology**	J Gastroenterol	0944-1174
**American Journal of Physiology-Gastrointestinal and Liver Physiology**	AM J Physiol-Gastr L	0193-1857
**Journal of Viral Hepatitis**	J Viral Hepatitis	1352-0504

Abbreviations: JCR, Journal Citation Reports from ISI Thomson Reuters

**Table 2:** Self-Citations and Impact Factors of Nine Top Gastroenterology and Hepatology Journals

Journals	2011	2010	2009	2008	2007	2006	2005
**Gastroenterology **							
IF	11.675	12.032	12.899	12.591	11.673	12.457	12.386
Self-Citations to Years Used in Impact Factor Calculation, No. (%)	420 (5)	454 (4)	398 (4)	429 (4)	423 (4)	491 (5)	506 (5)
Impact Factor without Self-Citations	11.155	11.433	12.347	12.003	11.077	11.779	11.655
**Hepatology **							
IF	11.665	10.885	10.84	11.355	10.734	10.446	9.792
Self-Citations to Years Used in Impact Factor Calculation, No. (%)	707 (7)	681 (7)	805 (10)	619 (8)	554 (8)	647 (10)	499 (8)
Impact Factor without Self-Citations	10.786	10.019	9.751	10.435	9.814	9.373	8.98
**GUT **							
IF	10.111	10.614	9.357	9.766	10.015	9.002	7.692
Self-Citations to Years Used in Impact Factor Calculation, No. (%)	153 (4)	159 (4)	174 (4)	202 (4)	174 (3)	196 (4)	234 (5)
Impact Factor without Self-Citations	9.696	10.188	8.969	9.343	9.642	8.621	7.278
**J Hepatol **							
IF	9.264	9.334	7.818	7.056	6.642	6.073	4.931
Self-Citations to Years Used in Impact Factor Calculation	267 (6)	232 (5)	222 (6)	233 (6)	191 (5)	196 (6)	241 (9)
Impact Factor without Self-Citations	8.681	8.807	7.305	6.574	6.285	5.686	4.47
**AM J Gastroenterol**							
IF	7.282	6.882	6.012	6.444	6.101	5.608	5.116
Self-Citations to Years Used in Impact Factor Calculation, No. (%)	268 (6)	315 (7)	354 (8)	336 (7)	309 (7)	327 (9)	270 (7)
Impact Factor without Self-Citations	6.821	6.392	5.484	5.961	5.656	5.102	4.73
**Liver Int **							
IF	3.824	3.84	2.987	2.908	2.559	2.344	1.766
Self-Citations to Years Used in Impact Factor Calculation, No. (%)	84 (5)	173 (12)	75 (7)	56 (5)	50 (6)	40 (6)	19 (6)
Impact Factor without Self-Citations	3.599	3.364	2.752	2.743	2.403	2.184	1.655
**J Gastroenterol **							
IF	4.16	3.61	2.909	3.117	2.052	1.927	1.532
Self-Citations to Years Used in Impact Factor Calculation, No. (%)	70 (5)	69 (6)	65 (7)	52 (5)	57 (9)	47 (7)	43 (7)
Impact Factor without Self-Citations	3.931	3.38	2.691	2.953	1.866	1.784	1.413
**AM J Physiol-Gastr L**							
IF	3.431	3.522	3.258	3.587	3.761	3.681	3.472
Self-Citations to Years Used in Impact Factor Calculation, No.	128 (6)	166 (8)	164 (7)	164 (7)	216 (9)	181 (8)	185 (9)
Impact Factor without Self-Citations	3.197	3.239	3.006	3.331	3.394	3.363	3.13
**J Viral Hepatitis **							
IF	4.088	3.502	3.348	3.326	2.971	3.29	2.55
Self-Citations to Years Used in Impact Factor Calculation, No. (%)	68 (7)	38 (4)	36 (4)	35 (4)	30 (4)	27 (4)	16 (4)
Impact Factor without Self-Citations	3.788	3.336	3.204	3.182	2.825	3.13	2.443

## 4. Results

**Figure 1:**
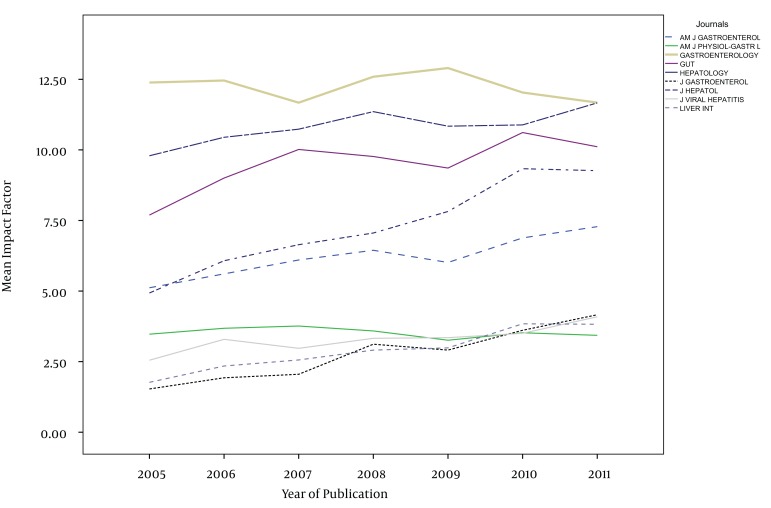
Trends of Impact Factors of Top Nine Journals Between 2005 and 2011

**Figure 2:**
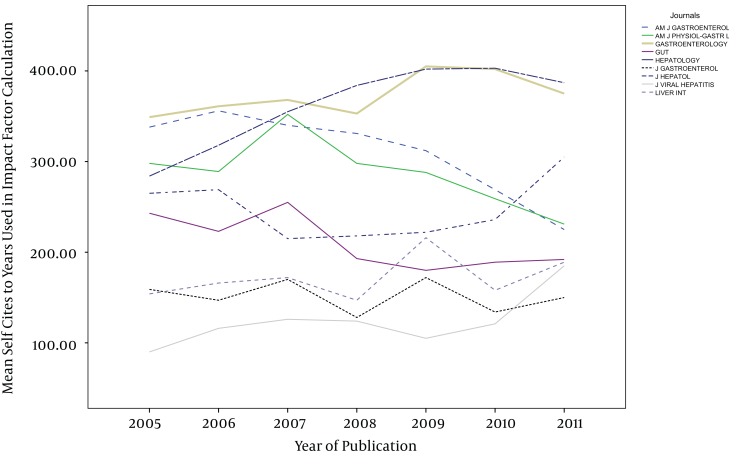
Trends of Number of Published Articles in Top Nine Journals Between 2005 and 2011

**Figure 3:**
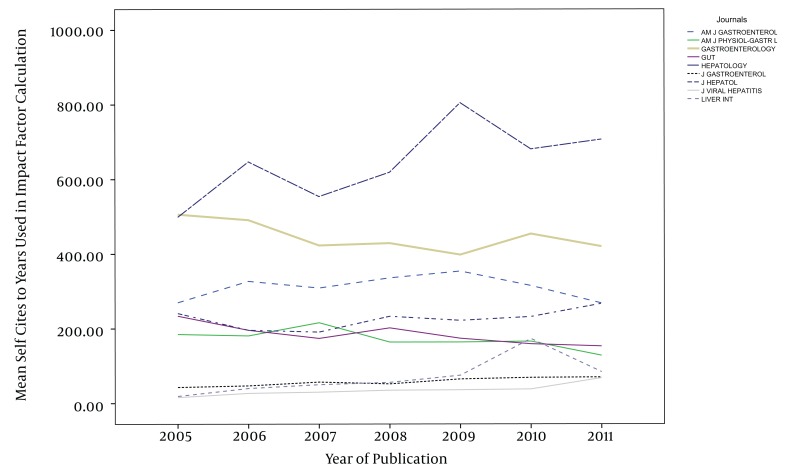
Trends of Self-Citations of Top Nine Journals Between 2005 and 2011

**Figure 4:**
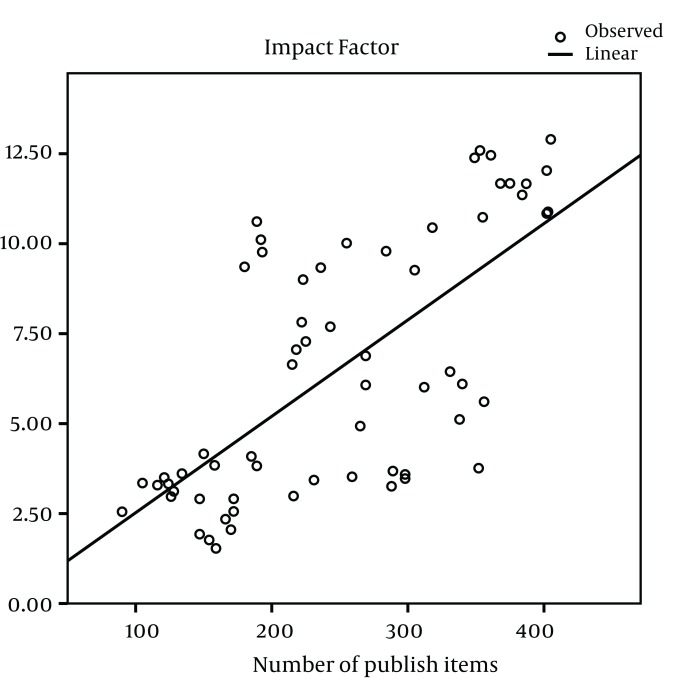
Regression Analysis of Impact Factor and Total Number of Published Articles in Top Nine Journals During 2005 to 2011 ^a^ ^a^ Mean ± STD of number of published items and impact factor tested using Pearson correlation coefficient. Correlation was significant at 0.01 levels (2-tailed). The curve fitting based on regression analysis on the number of published items as an independent variable: Unstandardized coefficients were as B: 0.27, Std. Error: 0.004, Beta: 0.688, and Sig: 0.000.

**Figure 5:**
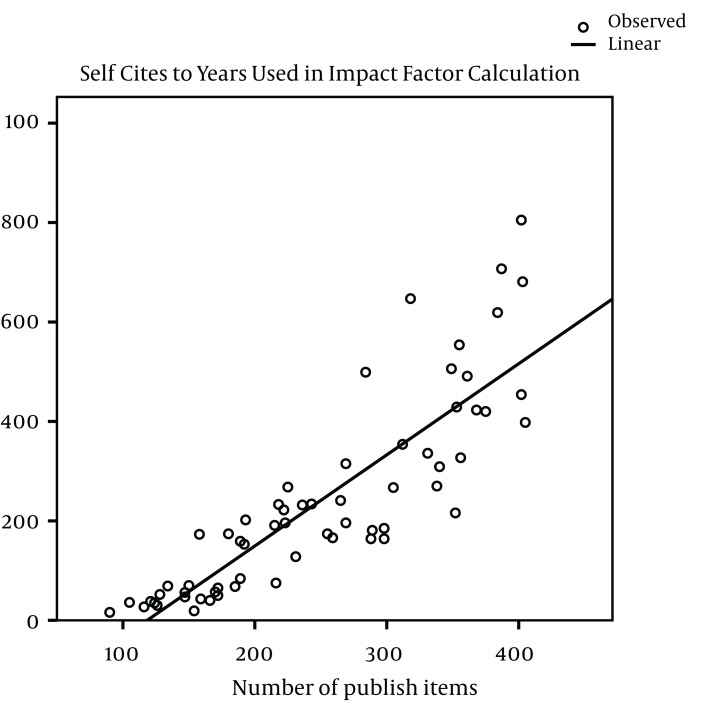
Regression Analysis of Self-Citation and Total Number of Published Articles in Top Nine Journals During 2005 to 2011 ^a^ ^a^The curve fitting based on regression analysis on the number of published items as an independent variable: Unstandardized coefficients were as B: 1.83, Std. Error: 0.139, Beta: 0.861, and Sig: 0.000. Mean ± STD of the number of self-citations to the years used in the calculation of impact factor and the number of total publications tested using Pearson correlation coefficient. Correlation was significant at 0.01 levels (2-tailed).

**Figure 6:**
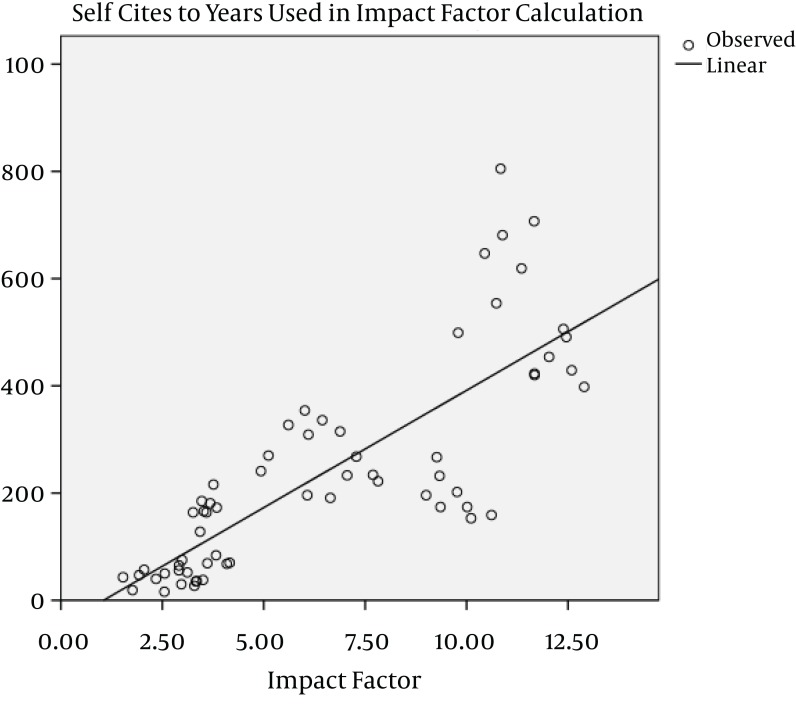
Regression Analysis of Self-Citation and Impact Factor of Top Nine Journals During 2005 to 2011 ^a^ ^a^ The curve fitting based on regression analysis on the number of published items as an independent variable: Unstandardized coefficients were as B: 43.7, Std. Error: 4.1, Beta: 0.80, and Sig: 0.000. Mean ± STD of impact factor and number of self-citations to the years used in the calculation of impact factor tested using Pearson correlation coefficient. Correlation was significant at 0.01 levels (2-tailed).

The impact factors in most of the journals have been increased gradually during the study years, except in AM J PHYSIOL-GASTROL and GASTROENTEROLOGY (Figure 1). Five out of nine journals increased and four journals decreased the number of their publications from 2005 to 2011. The maximum of increase in number of publication was seen in J VIRAL HEPATITIS (90 in 2005 to 185 in 2011) (Figure 2). During the past seven years of publishing, self-citations of top nine journals had almost no changes, except in HEPATOLOGY which were increased from 499 in 2005 to 707 in 2011 (Figure 3). The impact factors of journals (6.5 ± 3.5) were positively correlated with the total number of publications (248.6 ± 91.7) (R: 0.688, P < 0.001) (Figure 4). The self-citation rate (238.73 ± 195.317) was highly correlated with the total number of publication in each journal (248.6 ± 91.7) (R: 0.861, P < 0.001) (Figure 5). There was a positive correlation between impact factor (6.5 ± 3.5) and self-citations to the years used in the calculation of impact factor (238.73 ± 195.317) using Pearson correlation coefficient (R: 0.80, P < 0.001) (Figure 6). On the other hand, impact factor without self-citations (6.08 ± 3.3) and number of published items (248.6 ± 91.7) had a correlation as R: 0.672 and P < 0.001. In the curve fitting based on regression analysis on the number of published items as an independent variable, the unstandardized coefficients were as B: 0.025, Std. Error: 0.003, Beta: 0.672, and Sig: 0.000 (data were not shown).

## 5. Discussion

During the recent years and thanks to developments in internet technology and creation more facilities for authors to publish their articles, impact factors of journals have increased gradually that may be due to the increase in the number of readers. More user-friendly journal websites lead to more readers as well as more citations. Consequently, increasing in impact factors of journals motivates authors to publish their articles in those journals (11). Increase in the number of publication in each journal is correlated with IF. Therefore, not only more publication in a journal is considered as a potential risk, but also it should be noted as an important advantage for the journal. Editors as well as publishers should move towards increasing the number of their articles per year without any hesitation about the decreasing of IF. Although one of the utmost important factors in IF calculation is the total number of publications, it seems not to be a negative parameter for the journal at all (12). The self-citation rate was positively correlated with the number of publications. As a principle, editors should avoid increasing the self-citations in their journals but in some cases, authors expand it due to the tendency for increasing the chance of publishing their manuscripts or even increasing their H-index. Since the editors cannot directly oblige the authors to modify references or citations, it may lead to an increase in the total number of self-citations. The number of publications can directly affect on the self-citation and IF, too. Based on an incorrect opinion, some authors believe that adding some references from a favorite journal can increase the chance of publishing for them which in fact may increase the self-citation rate (13). Similar to our results, both Mehrad et al. and Triaridis et al. have shown that the number of journal self-citation was directly correlated with the number of published articles (14, 15).

According to our results, the self-citation rate has a direct effect on IF. One of the reasons is the authors rule by which they refer more to their own previous publications in the same journal (16). Moreover, the self-citation can increase the visibility of an article, so it may increase its citation by other authors, and subsequently increase the IF of journal (17). Another key rule of increasing IF dependency to self-citation is related to the editors that interfere with the review process and oblige the authors to include some extra citations from the journal which is strongly denied by committee of publication in ethics. Although considerable number of self-citation was detected in the mentioned journals, the self-citation ratio to the total number of citations was lower than that was in other journals because of high total number of publications per year (5). Fassoulaki et al. also revealed a significant correlation between self-citing rate and IF of a journal (5). Similarly, Vitzthum K et al. reported that the self-citation in some countries was increased (18). In a study by Gami et al., the effect of article type on self-citation was evaluated and it was shown that self-citation to the original articles seems to be twice compared to review articles. In addition, the self-citation rate varied among different journals in terms of their subjects. For instance, Gami et al. reported that one fifth (20%) of citations in the diabetes literatures were self-citations (19), and Fassoulaki et al. indicated that the self-citations in the anaesthesia journals were less than 30% of all citations (5). Based on Thomson Reuters report, about 80% of listed journals in JCR have self-citation rates of lower than, or equal to 20% (8). However, it mainly depends on the subject category of the journals (7). If a journal frequently exceeds the normal rate of self-citation and ISI detects that the journal is using self-citation improperly, the journal IF won’t be published and the journal name will be deselected by Web of Science (8). In conclusion, the number of articles and self-citation have definite effects on IF of a journal and because IF is the most prominent criterion for journal’s quality measurement, it would be a good idea to consider factors affecting on IF such as self-citation.

## References

[1] Wikipedia. Impact factor.

[2] Garfield E (2006). The history and meaning of the journal impact factor.. JAMA.

[3] Hoeffel C (1998). Journal impact factors.. Allergy.

[4] Amin M, Mabe M (2000). Impact factors: use and abuse.. Perspectives in publishing.

[5] Fassoulaki A, Paraskeva A, Papilas K, Karabinis G (2000). Self-citations in six anaesthesia journals and their significance in determining the impact factor.. Br J Anaesth.

[6] Web of Knowledge. Journal Citation Reports.

[7] Gloninger MH, Thomson I The ISI® Database: The Journal Selection Process..

[8] Testa J (2010). The Thomson Reuters journal selection process.. Retrieved February..

[9] Ha TC, Tan SB, Soo KC (2006). The journal impact factor: too much of an impact?. Ann Acad Med Singapore.

[10] Kao JH, Wu NH, Chen PJ, Lai MY, Chen DS (2000). Hepatitis B genotypes and the response to interferon therapy.. J Hepatol.

[11] Kovacic N, Misak A (2004). What can be learned from impact factor of Croatian Medical Journal, 1994-2003?. Croat Med J.

[12] Falagas ME, Alexiou VG (2008). The top-ten in journal impact factor manipulation.. Arch Immunol Ther Exp (Warsz)..

[13] Rad AE, Shahgholi L, Kallmes D (2012). Impact of self-citation on the H index in the field of academic radiology.. Acad Radiol.

[14] Mehrad J, Goltaji M Correlation between Journal Self-citation and Impact Factor in ISC's PJCR Agriculture and Veterinary Science Journals during..

[15] Triaridis S, Kyrgidis A (2010). Peer review and journal impact factor: the two pillars of contemporary medical publishing.. Hippokratia.

[16] Anseel F, Duyck W, De Baene W, Brysbaert M (2004). Journal impact factors and self-citations: implications for psychology journals.. Am Psychol.

[17] Fowler JH, Aksnes DW (2007). Does self-citation pay?. Scientometrics.

[18] Vitzthum K, Spallek M, Mache S, Quarcoo D, Scutaru C, Groneberg DA (2010). Cruciate ligament: density-equalizing mapping and scientometrics as a measure of the current scientific evaluation.. European Journal of Orthopaedic Surgery & Traumatology.

[19] Gami AS, Montori VM, Wilczynski NL, Haynes RB (2004). Author self-citation in the diabetes literature.. CMAJ.

